# Successful emergency transcatheter aortic valve replacement in the setting of hemodynamically unstable prosthetic aortic valve stenosis

**DOI:** 10.34172/jcvtr.2022.04

**Published:** 2022-03-07

**Authors:** Muhammad Ishaq, Muhammad Shoaib Khan, Amir Shahbaz, Jayanth G Vedre, Alon Yarkoni, Rhianna Malovrh, Juan Mesa

**Affiliations:** ^1^Department of Internal Medicine, Marshfield Clinic Health System, Marshfield, WI, USA; ^2^Department of Medicine, Sheikh Zayed Hospital, Lahore, Pakistan; ^3^Department of Critical Care, Marshfield Clinic Health System, Marshfield, WI, USA; ^4^Department of Cardiovascular Disease, UHS Wilson Medical Center, Johnson City, NY, USA; ^5^Department of Cardiovascular Disease, Marshfield Clinic Health System, Marshfield, WI, USA

**Keywords:** Prosthetic Aortic Valve Stenosis, Acute Decompensated Heart Failure, Transcatheter Aortic Valve Replacement

## Abstract

FDA approved transcatheter aortic valve replacement (TAVR) for the treatment of symptomatic aortic valve (AV) stenosis. Recent evidence reveals that TAVR is the treatment of choice in most patients with AV stenosis who are at high risk for surgical aortic valve replacement (SAVR). Per AHA guidelines, repeat valve replacement has been recommended for bio-prosthetic AV stenosis. Urgent TAVR for hemodynamically unstable patients with prosthetic AV stenosis is not supported by significant scientific data. However, there have been a few cases reported on emergency TAVR procedures in hemodynamically unstable patients with severe native AV stenosis. We are reporting a unique case of successful emergency TAVR in a hemodynamically unstable patient, who had severe symptomatic bio-prosthetic AV stenosis at the time of presentation.

## Introduction

 With the advent of TAVR, patients with symptomatically severe AS started benefiting in terms of symptoms and mortality.^1,2,3^ The indications extended from other patient populations, including valve-in-valve (ViV) treatments for bioprosthetic aortic valve degeneration.^4^ Prior studies have revealed excellent and promising outcomes of TAVR up to five years post-implantation with preliminary studies reporting evidence of early transcatheter valve degeneration approximately eight years following implantation.^5^ The ViV TAVR for patients with prosthetic valve degeneration appears to be a safe and effective alternative to surgery,^6^ and its role as a treatment of choice in severely deteriorated prosthetic aortic valve has not been well established.^4^ However, published data have shown that emergency TAVR could be a potentially feasible and safe treatment option for prosthetic AV degeneration due to stenosis.^7^ Our report highlights a unique case of prosthetic AV deterioration with stenosis, presenting with cardiogenic shock, and successfully treated with emergency TAVR.

## Case Description

**Figure 1:**
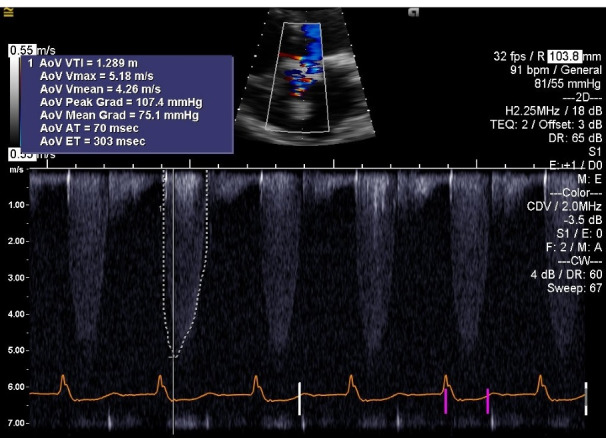
Pre-TAVR ViV echocardiogram; Color Doppler M-mode. Patient has 27 mm St. Jude bio-prosthetic aortic valve. AV peak gradient (AoV Peak Grad) is 107.4 mm Hg, AV mean gradient (AoV Mean Grad) is 75.1 mm Hg and peak velocity (AoV Vmax) is 5.18 m/s AoV, Aortic Valve

 A 65-year-old male patient with past medical history significant for coronary artery disease (CAD), and aortic valve stenosis. He underwent coronary artery bypass graft (CABG) surgery, and aortic valve replacement with a Saint Jude Epic # 27 bio-prosthesis, 11 years prior to admission. He had severe oxygen dependent COPD, chronic kidney disease stage IV, renal cell carcinoma, and history of GI bleed. He was admitted to the hospital with progressive severe dyspnea, and hypotension. At presentation, the patient’s blood oxygen saturation was 85-87% on supplemental oxygen, blood pressure of 70/50 mm Hg, and signs suggestive of fluid overload. Chest X ray revealed pulmonary congestion with bilateral pleural effusion. Laboratory tests showed an elevated serum BNP of 5600 pg/mL (Normal value = 0-80 pg/mL). Electrocardiogram (EKG) was suggestive of sinus rhythm with right bundle branch block (RBBB) and right axis deviation. Echocardiogram (Figure 1) revealed severe stenosis of the bio-prosthetic AV with a peak gradient of 107.4 mm Hg, mean gradient of 75.1 mm Hg, peak velocity of 5.18 m/s, AV area of 0.62 cm^2^, and preserved left ventricular ejection fraction (LVEF) of 60%. As the patient was in cardiogenic shock, he required vasopressors, including, norepinephrine, phenylephrine, and vasopressin. For respiratory support, he required Bi-level positive airway pressure (BiPAP). Continuous veno-venous hemodialysis (CVVHD) was utilized to treat acute worsening of chronic kidney disease and fluid overload.

**Figure 2:**
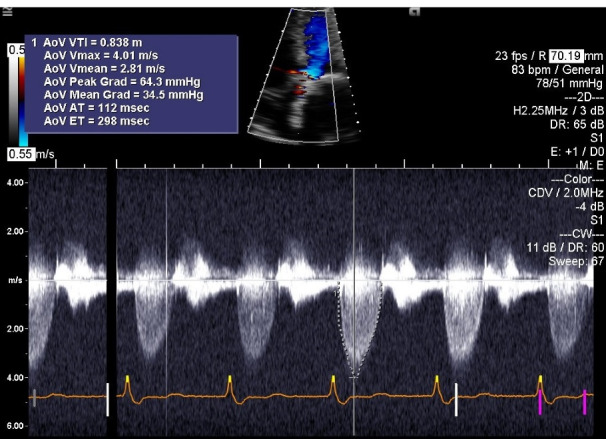
1 week post-TAVR echocardiogram; Color Doppler M-mode. Patient has 29mm Medtronic Evolut Pro bioprosthetic transcatheter aortic valve. AV peak gradient (AoV Peak Grad) is 64.3 mm Hg, AV mean gradient (AoV Mean Grad) is 34.5 mm Hg and peak velocity (AoV Vmax) is 4.01 m/s AoV, Aortic Valve

**Figure 3:**
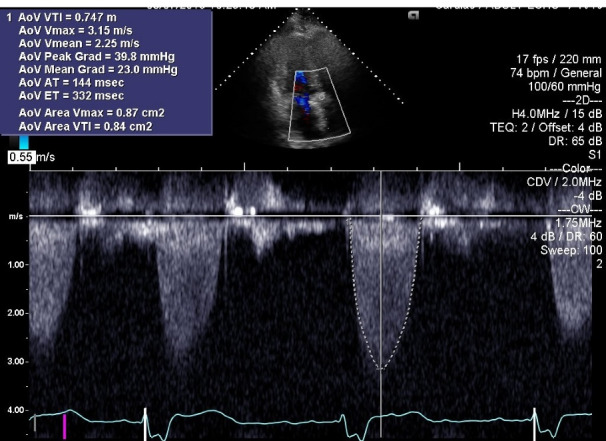
Post-TAVR echocardiogram at 5 months interval; Color Doppler M-mode. Prosthetic AV peak gradient of 39.8 mm Hg, mean gradient of 23.0 mm Hg, and peak velocity of 3.15 m/s. AoV, Aortic Valve

 His acute decompensated heart failure was believed to be secondary to structural bio-prosthetic valve degeneration with associated critical stenosis and moderate regurgitation. Because of unstable hemodynamics, multiple comorbidities, high logistic Euro- score II of 67.0% and Society of Thoracic Surgery Risk (STS) score of 46%, the patient was deemed to be a poor surgical candidate for re-do surgical intervention. Therefore, he underwent immediate and successful TAVR with a ViV procedure with a 29 mm Medtronic Evout –R, using transfemoral approach with conscious sedation without complications. Follow up echocardiogram (Figure 2) one week post-procedure revealed prosthetic AV peak gradient of 64.3 mm Hg, mean gradient of 34.5 mm Hg, peak velocity of 4.01 m/s, and LVEF of 60%. The patient’s symptoms improved remarkably and he was discharged from the hospital after an extended stay of around 3 weeks. The patient had past medical history of CAD and was already on daily oral aspirin without any indication for concurrent dual antiplatelet therapy or any indication for anticoagulation. Another follow up echocardiogram at 5 months interval (Figure 3) revealed prosthetic AV peak gradient of 39.8 mm Hg, mean gradient of 23.0 mm Hg, and peak velocity of 3.15 m/s.

## Discussion

 TAVR is currently the “gold-standard” treatment for high to intermediate-risk elderly patients with severe AS.^7^ Because of the limited therapeutic options, prosthetic valve AS with decompensated heart failure is an exceedingly challenging scenario. Often, some of these patients are not surgical candidates due to their multiple comorbid medical conditions and precarious hemodynamic state. Emergency TAVR, especially in reference to the aforementioned patient population, may serve as a reasonable treatment option.^7^ Even though emergency TAVR may have high immediate procedural and 30-day mortality compared to emergency balloon aortic valvuloplasty, it is shown to have been associated with lower mortality at 2-years follow-up.^8^ Frerker and et al. studied outcomes of TAVR in patients with cardiogenic shock due to acute decompensated heart failure caused by severe AS. Interestingly, among 30-day survivors, one-year survival did not differ between emergency and elective TAVR groups (89.6% vs. 88.9%).^9^ Kim H and et al. reported a case of successful emergent TAVR in the setting of acute decompensated heart failure (and cardiorenal syndrome) from severe AS. They concluded that emergent TAVR is a relatively safe treatment option in those who cannot undergo surgery.^7^ Given the limited number of options for the management of acutely decompensated prosthetic valve AS patients, emergent TVAR could represent a new avenue of treatment, especially in those elderly patients with a significant risk of perioperative complications requiring urgent stabilization. However, the outcomes may vary based on the treatment center expertise with the TAVR. In one German study, patients with cardiogenic shock and severe prosthetic aortic stenosis requiring emergent TVAR (TAVI) had a 30-day mortality of 19% compared to 5% in those without cardiogenic shock. Even though 19% is quite high, it is still less than 26% mortality associated with conventional aortic valve repair in such a population.^10,11^

 Our case and other emerging data indicate that TAVR is a promising option in patients with prosthetic aortic valve stenosis and decompensated heart failure. It is high time for a further large center prospective clinical study to establish the long-term outcomes in this patient population.

## Conclusion

 Emergency TAVR treatment for prosthetic AV stenosis in the setting of unstable hemodynamic clinical state and presence of multiple comorbidities can be a safe alternative to surgery in carefully selected patient population who cannot tolerate surgery. However, further studies will have to be performed to establish whether it can be superior or at-least comparable to alternative procedures such as balloon aortic valvuloplasty when it comes to immediate procedural and 30-day mortality.

## Acknowledgements

 We wish to thank Rimsha Areej (BFN) for editing the article.

## Funding

 This case report received no grant from any funding agency in the public, commercial or not-for-profit sectors.

## Ethical Approval

 In this case study, the patient confidentiality has been strictly preserved.

## Competing interest

 There was no conflict of interest.
